# Progression of disease within 24 months (POD24) in multiple myeloma implicates poor prognosis and limitations of current prediction models for POD24

**DOI:** 10.1038/s41598-024-73822-w

**Published:** 2024-10-01

**Authors:** Yongqin Cao, Yingying Gong, Qingqing Wang, Jun Xia, Xin Zhou, Chao Sun

**Affiliations:** Department of Hematology, Wuxi People’s Hospital, Wuxi Medical Center, The Affiliated Wuxi People’s Hospital of Nanjing Medical University, Nanjing Medical University, 299 Qingyang Road, Wuxi, Jiangsu Province China

**Keywords:** Multiple myeloma, POD24, Prognostic analysis, Prognostic model, Prognostic factor, Haematological cancer, Cancer, Outcomes research

## Abstract

Multiple myeloma (MM) is a common hematological malignancy, and its prognostic factors have been extensively studied. Progression of disease within 24 months (POD24) suggests a poor prognosis in many malignancies, but is rarely mentioned in MM. This study aimed to investigate the prognostic value of POD24 in MM and risk factors of POD24, and to evaluate the predictive value of existing MM prognostic models for POD24. The research retrospectively analyzed the clinical data of MM patients and found that the occurrence of POD24 is an independent prognostic factor affecting overall survival in MM, while non-transplantion and genetic abnormality are independent risk factors for the occurrence of POD24. The existing prognostic models are not effective in predicting POD24. Therefore, it’s still necessary to explore a prognostic model that can predict POD24 more accurately.

## Introduction

Multiple myeloma (MM) is a heterogeneous hematological malignancy. While therapeutic advancements have significantly improved patient prognosis in recent years, a cure remains elusive^1^. Consequently, forecasting outcomes in MM remains a crucial topic in ongoing research. For example, in recent years, new staging systems such as Mayo Additive Staging System (MASS)^2^, the Second Revision of the International Staging System (R2-ISS)^3^ and Myeloma Prognostic Score System (MPSS)^4^ have been proposed internationally, all of which comprehensively consider tumor loading factors and cytogenetics, providing a new reference for better judging the prognosis of multiple myeloma. However, the construction of these prognostic models is based on the static parameters of patients at the first diagnosis, and lacks the dynamic evaluation parameters of patients’ curative effect after treatment.

Progression of disease within 24 months (POD24) emerged as an independent prognostic factor initially in follicular lymphoma^5^ and subsequently demonstrated its value in various malignancies. However, the impact of POD24 on MM prognosis has not been adequately investigated. This study, therefore, aimed to: (1) analyzed the effect of POD24 on prognosis in 473 MM patients, (2) explore factors in influencing POD24 occurance, and (3) evaluate the predictive accuracy of existing MM prognostic models for POD24.

## Materials and methods

### Ethics statement

This study followed the Helsinki declaration. All participants signed an informed consent form and this study was approved by the ethics committee of the Wuxi People’s Hospital, Wuxi Medical Center, Nanjing Medical University (Registration number: KY24002).

### Patients

A total of 526 newly diagnosed MM patients were initially included in this study. After excluding patients with missing initial diagnosis data, those who were not regularly admitted for treatment, those without disease progression within 24 months of follow-up, and those not followed for at least 24 months, a final cohort of 473 patients were retrospecyively analyzed.

All patients in this group underwent peripheral blood and bone marrow cytology examinations, strictly adhering to the International Myeloma Working Group (IMWG)^6^,diagnostic criteria. Among them, 238 patients recived fluorescence in situ hybridization (FISH) testing. Regarding treatment, 345 patients recived bortezomib-based therapy, which mainly included bortezomib + adriamycin + dexamethasone, bortezomib + pirarubicin + dexamethasone, bortezomib + thalidomide + dexamethasone, and bortezomib + lenalidomide + dexamethasone. The remaining 128 patients recived conventional chemotherapy, including vinnestine + adriamycin + dexamethasone, thalidomide + liposomal doxorubicin + dexamethasone, and liposomal doxorubicin + vinnestine + dexamethasone. All patients subsequently received maintenance therapy with a proteasome inhibitor. Age, sex, β2-microglobulin (β2-MG), ISS stage, lactate dehydrogenase (LDH) levels, serum creatinine, serum calcium and hemoglobinlevels were recorded for all MM patients before their initial chemotherapy. Overall survival (OS) and progression-free survival (PFS) were calculated.

### Staging standard

**International Staging System (ISS)** Stage I: Serum β2-MG < 3.5 mg/L and albumin ≥ 35 g/L; stage II: neither stage I nor stage III; stage III: β2-MG ≥ 5.5 mg/L.

**Revised International Staging System (R-ISS)** Stage I: ISS I with normal LDH, no critical cytogenetic markers; stage II: neither stage I nor stage III; stage III: ISS III with critical cytogenetic markers or elevated LDH. Critical cytogenetic markers include del (17p), t (4; 14) and t (14; 16).

**Mayo Stratification for Myeloma and Risk-adapted Therapy 3.0 (mSMART3.0)** High-risk group: merged t(4;14), t(14;16), t(14;20), 1q21 amplification, 17p deletion/mutation, R-ISS stage III, increased S stage plasma cell index, and high-risk gene expression profile. Standard-risk group: merged trisomies, t(11,14), t(6,14), or no other cytogenetic abnormalities.

**MASS** 1 point awarded for: high-risk IgH translocation (t(4;14), t(14;16), or t(14;20)), 1q gain/amplification, chromosome 17 abnormality, ISS III, and elevated LDH. Patients were divided into stages I (0 points), II (1 point) and III (2 + points).

**R2-ISS** 1 point awarded for: chromosome 17 abnormality, ISS II, t(4;14), and elevated LDH; ISS III: 1.5 points; 1q gain/amplification: 0.5 points; stages I (0 points), II (0.5_1 point), III (1.5_2.5 points), and IV (3_5 points).

### Follow-up visit

Follow-up primarily involved consulting outpatient and inpatient medical records, supplemented by telephone calls. All patients were followed until November 30, 2022, or death. OS is defined as the time from diagnosis to patient death or the end of follow-up. PFS is defined as the time from diagnosis to disease progression or disease-related death. POD24 was defined as disease progression occurring within 24 months of definitive diagnosis and initiation of treatment.

Efficacy was assessed using the IMWG criteria^6^. Disease progression was defined as any of the following: (1) An increase of serum M protein ≥ 25% (absolute increase ≥ 5 g/L) or an increase of M protein ≥ 10 g/L (baseline serum M protein ≥ 50 g/L). (2) Urinary M protein increased by ≥ 25% (absolute increase ≥ 200 mg/24 h). (3) In the absence of detectable serum or urine M protein, an increase of ≥ 25% with an absolute increase of > 100 mg/L in the difference between affected and non-affected FLC values. (4) Increase ≥ 25% with an absolute increase of ≥ 10% in the proportion of bone marrow plasma cells, if neither serum nor urine M protein nor serum FLC can be measured. (5) Increase from the lowest point in the sum of the product of the maximum vertical diameter of more than one measurable lesion by ≥ 50%; Increase of ≥ 50% in the long axis of an original lesion ≥ 1 cm. (6) Increase ≥ 50% (absolute value ≥ 200 cells per microliter required if only circulating plasma cells are used as measurable lesions). In this study, 158 patients met the above definition, and 315 patients did not develop POD24. The patients were divided into the POD24 group and the non-POD24 group according to the above definition of POD24 and disease progression.

### Statistical analysis

SPSS 26.0 software was used for statistical analysis. Kruskal-Wallis and Mann_Whitney methods were employed to compare continuous variables between groups. Chi-square tests assessed differences in categorical variables, with Fisher’s exact test applied when necessary. Kaplan_Meier analysis generated survival curves, and log-rank tests evaluatde survival differences (*p* < 0.05 cansidered statistically significant). Survival curves were plotted using GraphPad Prism 6.0. COX regression models were used for univariate and multifactorial analysis, with *P* < 0.05 considered statistical. Diagnostic prediction value calculations, ROC curve generation, and area under the curve (AUC) analysis were performed using SPSS 26.0 software, with *p* < 0.05 considered statistically significant.

## Results

### Comparison of clinical features and survival analysis between POD24 and non-POD24 groups

**Fig. 1:**
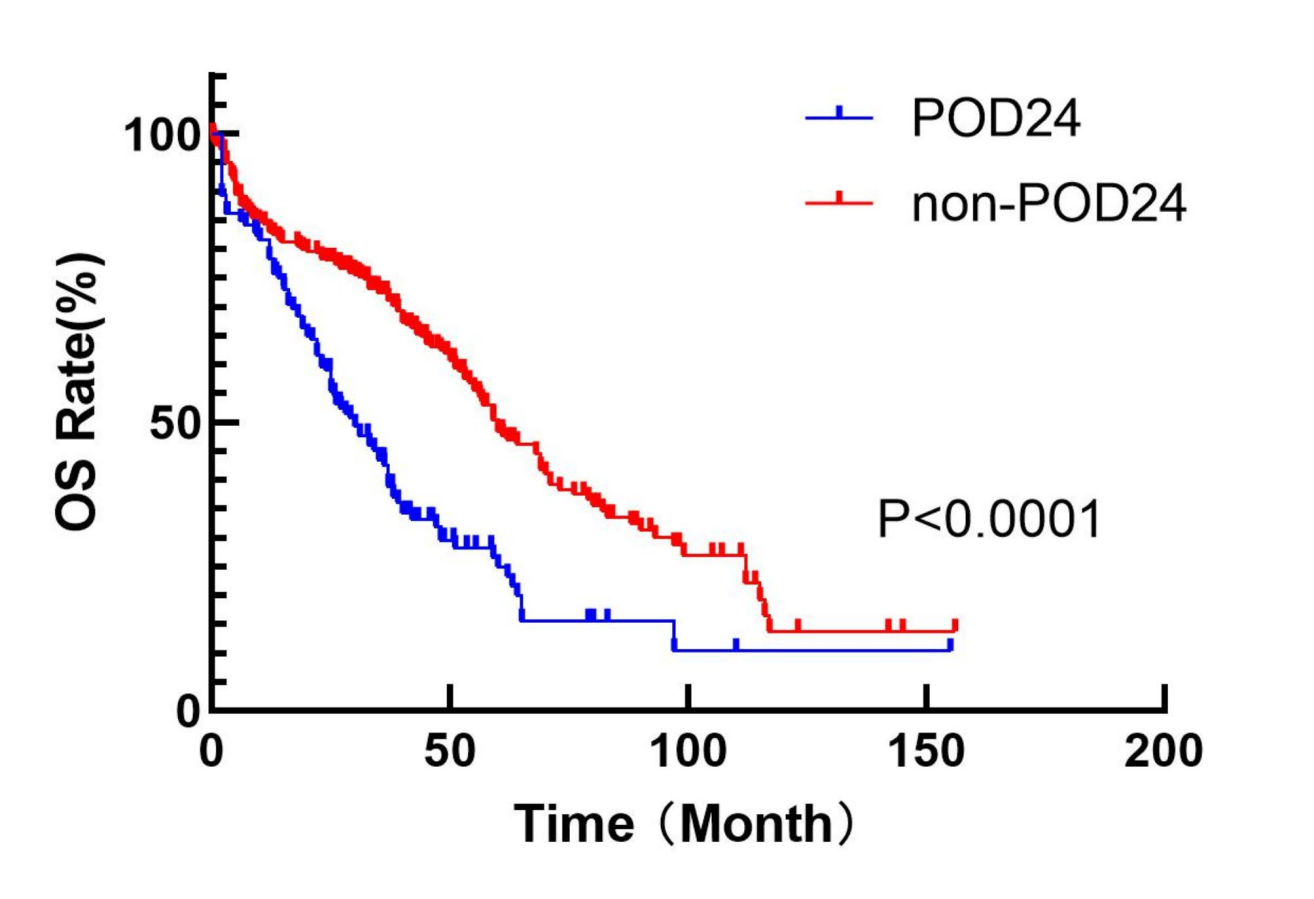
Comparison of OS between POD24 group and non-POD24 group in newly diagnosed MM patients.

**Fig. 2:**
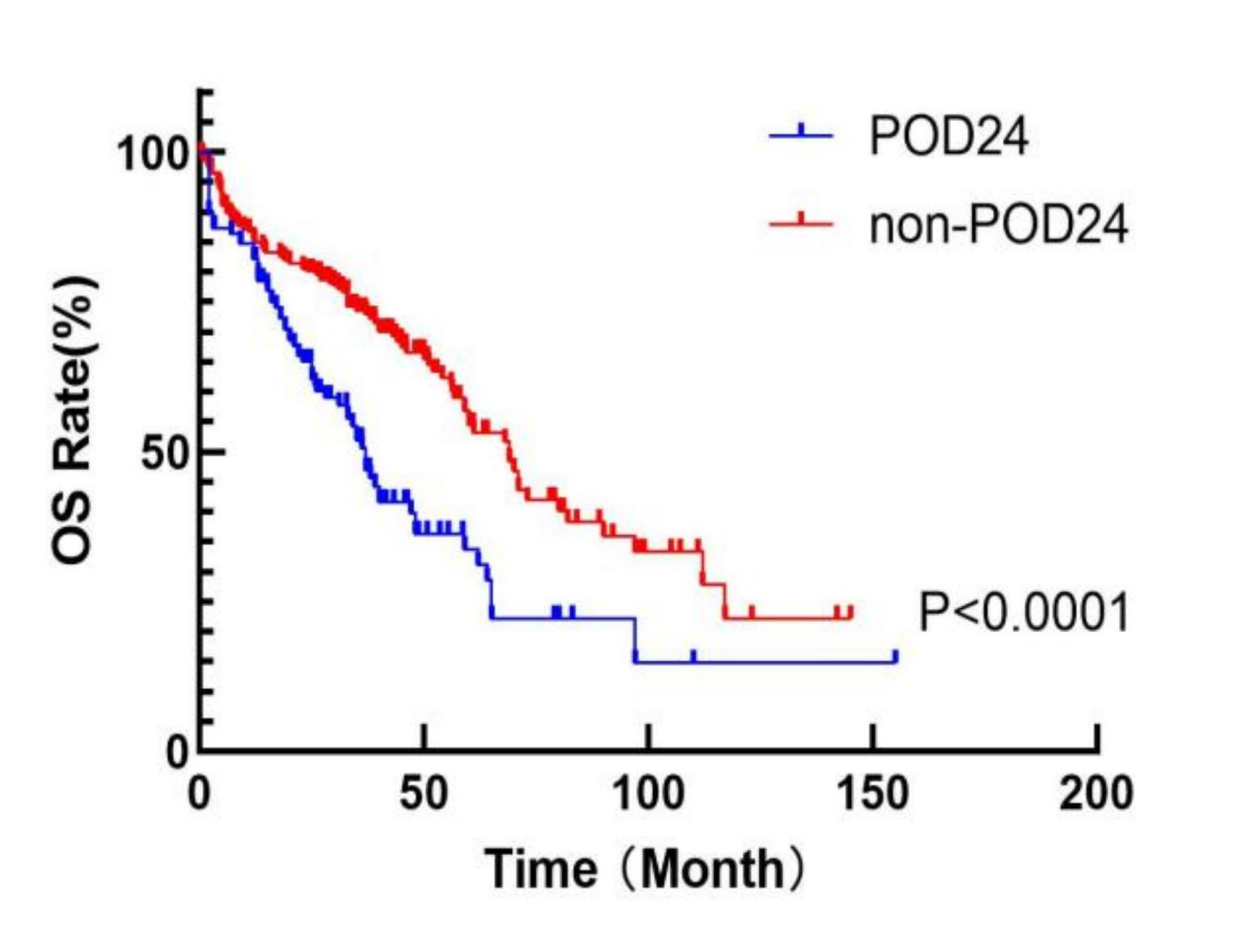
Survival curves of OS between POD24 and non-POD24 groups in MM patients treated with bortezomib-based therapy.

Table 1 presents the clinical parameters and a comparison of patients in the POD24 and non-POD24 groups. There were significant differences in hemoglobin, serum calcium ion concentration and chromosome information between two groups (*P* < 0.05). Survival analysis revealed statistically differences in OS between patients in POD24 and non-POD24 groups (POD24 group vs. non-POD24 group: median OS_30.0 (95% CI 23.2–36.8) vs. 60.0 (95% CI 51.8–68.2) months, *p* < 0.0001). This finding, illustrated in Fig. 1, indicated a poorer prognosis for patients in the POD24 group. Of the patients included in this study, 345 patients received bortezomib-based treatment. In order to further exclude possible bias caused by treatment regimens, the study further performed survival analysis between POD24 group and non-POD24 group in patients treated with bortezomib. The results showed that there was a significant difference in OS between the two groups (POD24 group vs. non-POD24 group: median OS: _37.0 (95% CI 31.7–42.3) vs. 69.0 (95% CI 60.3–77.7) months, *p* < 0.0001) (Fig. 2).

**Table 1:** Clinical characteristics of MM patients in POD24 group and non-POD24 group.

Clinical character		POD24 (*n* = 158)	Non-POD24 (*n* = 315)	*P*
Age		62.7(40–90)	61.4(24–85)	0.197
Gender	Male	92(58.2%)	171(54.3%)	0.399
	Female	66(41.8%)	144(45.7%)	
Hemoglobin (g/L)		85.1(37–169)	91(43–163)	0.017
Blood calcium (mmol/L)		2.37(1.38–5.35)	2.27(1.61–4.05)	0.030
Creatinine(umol/L)		164.1(38-1197.8)	166.7(30-1286.7)	0.904
β2 microglobulin (ug/L)		8.5(0.26–39.02)	9.32(0.1–343)	0.667
Lactate dehydrogenase(U/L)		195.1(50-1102)	194.4(25.4–4322)	0.987
ISS staging	I	23(14.6%)	47(14.9%)	0.331
	II	51(32.3%)	122(38.7%)	
	III	84(53.2%)	146(46.3%)	
Treatment	Contains bortezomib	118(74.7%)	227(72.1%)	0.545
	Without bortezomib	40(25.3%)	88(27.9%)	
R-ISS staging	I	18(11.4%)	38(12.1%)	0.910
	II	110(69.6%)	222(70.5%)	
	III	30(19.0%)	55(17.5%)	
Transplant state	Transplant	24(15.2%)	72(22.9%)	0.051
	Non-transplant	134(84.8%)	243(77.1%)	
Chromosomal	Normal	96(60.8%)	227(72.1%)	0.013
	Abnormal	62(39.2%)	88(27.9%)

### Univariate and multifactorial analysis of OS in newly diagnosed MM patients

According to the COX univariate analysis of OS in newly diagnosed MM patients in this study, several factors were associated with shorter OS (*p* < 0.05): age > 60 years (HR 1.898), hemoglobin < 100 g/L (HR 1.680), LDH > 245 U/L (HR 1.605), β2-MG > 3.5 mg/L (HR 2.456), creatinine > 177 µmol/L (HR 2.231), elevated serum Ca + > 2.75 mmol/L (HR 2.623), POD24 occurrence (HR 2.100), non-transplantion (HR 0.111), and treatment regimens without bortezomib (HR 0.617). Multivariate analysis confirmed that LDH > 245 U/L (HR 1.447), β2-MG > 3.5 mg/L (HR 1.728), creatinine > 177 µmol/L (HR 1.563), elevated serum Ca + > 2.75 mmol/L (1.576), treatment regimens without bortezomib (HR 0.638), non-transplantion (HR 0.171), and POD24 (HR 1.956) remained independent prognostic factors for OS (*p* < 0.05) (Table 2).

**Table 2:** Univariate and multifactorial analysis of OS in newly diagnosed MM patients.

Clinical character	Univariate Analysis		Multivariate Analysis	
	HR(95%CI)	*P* value	HR(95%CI)	*P* value
Age(>60)	1.898(1.455–2.477)	0.000	1.300(0.982–1.722)	0.067
Hemoglobin(< 100 g/L)	1.680(1.264–2.233)	0.000	1.053(0.763–1.454)	0.754
Lactate dehydrogenase (> 245U/L)	1.605(1.160–2.219)	0.004	1.447(1.033–2.027)	0.032
β2 -microglobulin (> 3.5 mg/L)	2.456(1.784–3.380)	0.000	1.728(1.203–2.484)	0.003
Creatinine(> 177 µmol/L)	2.231(1.704–2.922)	0.000	1.563(1.152–2.122)	0.004
Blood calcium (> 2.75 mmol/L)	2.623(1.830–3.760)	0.000	1.576(1.046–2.374)	0.030
POD24	2.100(1.631–2.704)	0.000	1.956(1.507–2.541)	0.000
Treatment (without bortezomib)	0.617(0.480–0.794)	0.000	0.638(0.490–0.831)	0.001
Chromosomal abnormality	1.248(0.940–1.657)	0.126		
Non-transplantion	0.111(0.052–0.235)	0.000	0.171(0.079–0.371)	0.000

### Univariate and multivariate analysis of POD24 in patients with MM

To further investigate the factors influencing POD24 occurrence, COX univariate and multivariate analyses were performed in MM patients. Univariate analysis showed that hemoglobin < 100 g/L (HR 1.694), LDH > 245 U/L (HR 1.620), β2-MG > 3.5 mg/L (HR 1.614), elevated serum Ca + > 2.75 mmol/L (HR 1.886), non-transplantion (HR 0.558) and chromosomal abnormalities (HR 1.665) were associated with an increased risk of POD24 (*p* < 0.05). Multivariate analysis revealed that non-transplantion (HR 0.521) and chromosomal abnormalities (HR 1.747) emerged as independent risk factors for POD24 (*p* < 0.05) (see Table 3).

**Table 3:** Univariate and multivariate analysis of POD24 in newly diagnosed MM patients.

Clinical character	Univariate Analysis		Multivariate Analysis	
	HR(95%CI)	*P* value	HR(95%CI)	*P* value
Age(>60)	1.344(0.972–1.857)	0.074		
Hemoglobin(< 100 g/L)	1.694(1.172–2.449)	0.005	1.395(0.920–2.115)	0.117
Lactate dehydrogenase (> 245U/L)	1.620(1.088–2.413)	0.018	1.435(0.956–2.155)	0.081
β2 -microglobulin (> 3.5 mg/L)	1.614(1.120–2.325)	0.010	1.155(0.758–1.760)	0.502
Creatinine(> 177 µmol/L)	1.079(0.735–1.583)	0.700		
Blood calcium (> 2.75 mmol/L)	1.886(1.166–3.051)	0.010	1.530(0.931–2.512)	0.093
Treatment (without bortezomib)	1.036(0.724–1.483)	0.845		
Chromosomal abnormality	1.665(1.209–2.293)	0.002	1.747(1.251–2.439)	0.001
Non-transplantion	0.558(0.361–0.862)	0.009	0.521(0.331–0.819)	0.005

### Efficacy of existing MM prognostic models in predicting POD24

**Fig. 3:**
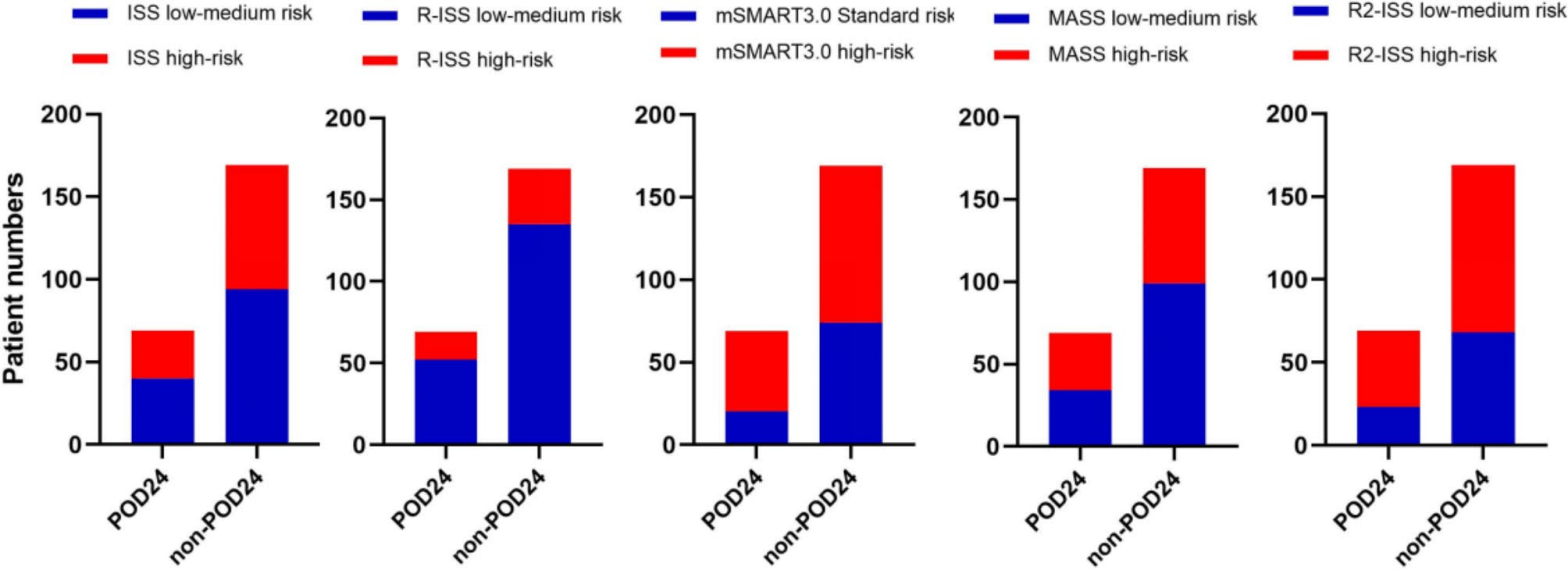
Staging of five prognostic models in patients with POD24 and non-POD24.

**Fig. 4:**
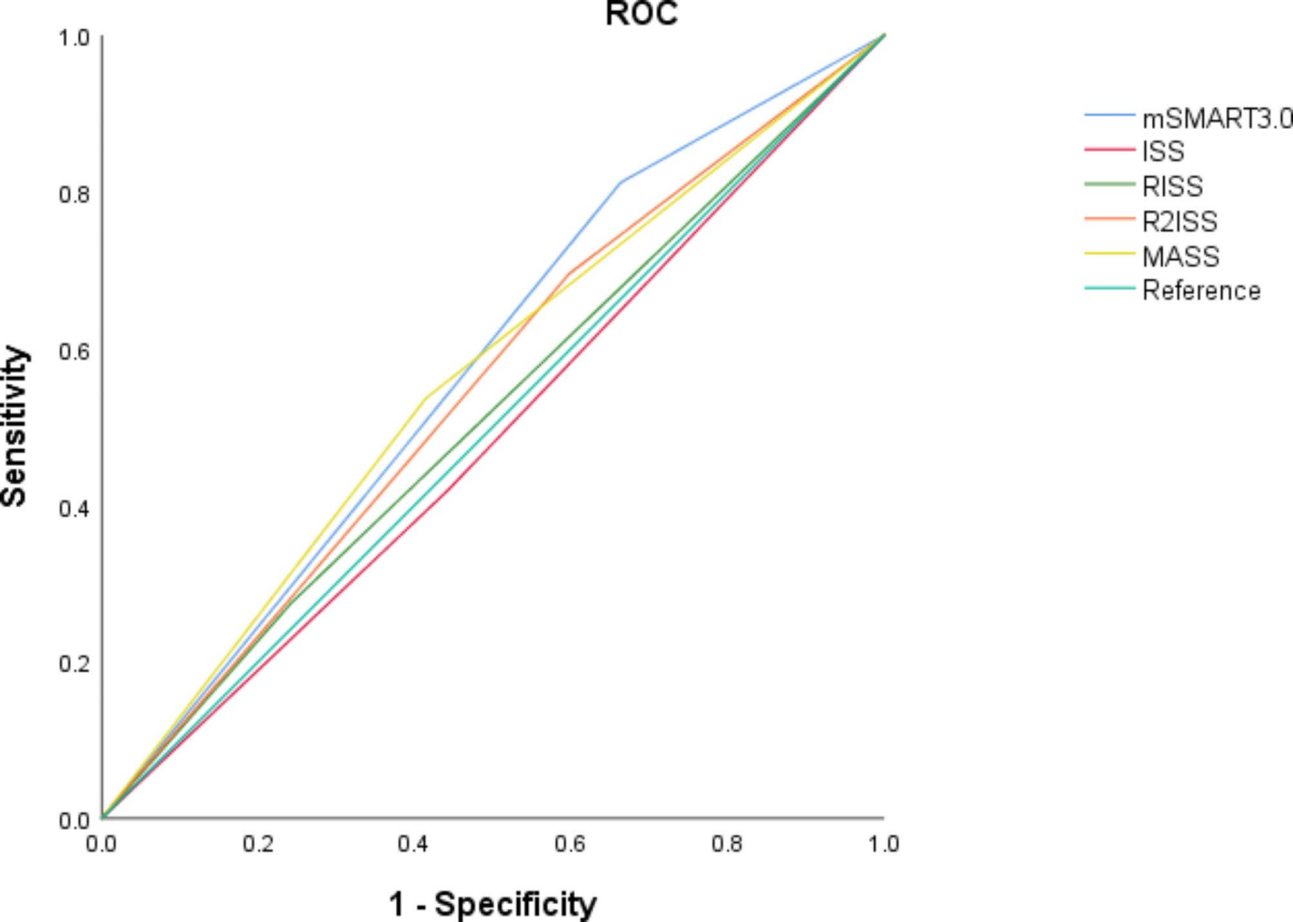
ROC curves of the five prognostic models for predicting POD24.

FISH detection was completed in 238 patients in this study, including 69 patients in the POD24 group and 169 patients without POD24 group. The stages of the two groups of patients were performed based on the existing MM prognostic model. The stages of each prognostic model for patients with POD24 and without POD24 group were shown in Fig. 3. Among the five models evaluated, mSMART3.0 had the highest sensitivity (81.2%) and R-ISS had the highest specificity (75.7%) for predicting POD24. However, the differences in predictive accuracy were not statistically significant. According to the ROC curve analysis, all the existing prognostic models had poor prediction efficiency for POD24 (see Table 4; Fig. 4).

**Table 4:** The predictive value of five prognostic models for POD24.

	Sensibility (%)	Specificity (%)	Accuracy (%)	Positive predictive value (%)	Positive predictive value (%)	AUC
mSMART3.0	81.2	33.7	47.5	33.3	81.4	0.574
ISS	42.0	55.6	51.7	27.9	70.1	0.488
R-ISS	27.5	75.7	61.8	31.7	71.9	0.516
R2-ISS	69.6	40.2	48.7	32.2	76.4	0.549
MASS	53.6	58.6	57.1	34.6	75.6	0.561

### Survival analysis between POD18 and POD18-24

**Fig. 5:**
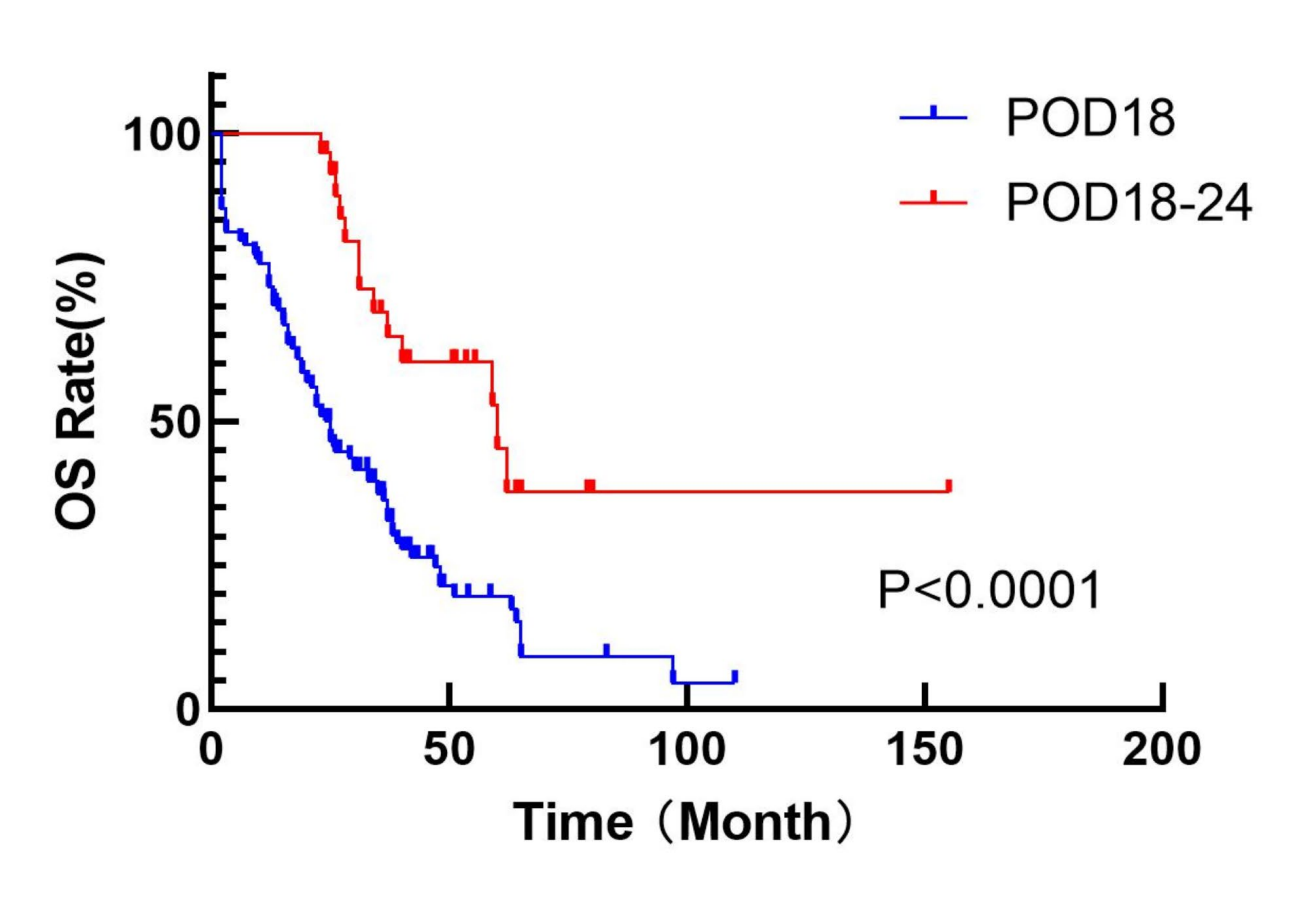
Comparison of OS between POD18 group and POD18-24 group.

We further grouped patients in the POD24 group into the progression of disease within 18 months (POD18) group and progression of disease within 18–24 months (POD18-24) group, and performed survival analysis. The results showed that there were statistically differences in OS between patients in POD18 and POD18-24 groups (POD18 group vs. POD18-24 group: median OS: _25.0 (95% CI 21.1–28.9) vs. 60.0 (95% CI 37.5–82.5) months, *p* < 0.0001) (Fig. 5).

## Discussion

While advancements in treatment have greatly improved MM prognosis, it remains an incurable malignant disease^1,7^. Studies have shown that elevated β2-microglobulin, LDH, serum calcium, and creatinine correlate with poor prognosis^8,9^. These markers suggest aggressive tumor activity, bone tissue invasion, or impaired kidney function, leading to shorter OS. Furthermore, cytogenetic abnormalities such as 1q amplification, del(17p), t(4; 14), and p53 mutations are established poor prognostic factors^1^. Prognostic models based on the above parameters, such as ISS and R-ISS, indeed provide a certain reference for the prognosis of newly diagnosed MM patients. However, in the actual clinical situation, in addition to high-risk patients, the prognosis of some low-risk patients also shows certain heterogeneity, indicating that the initial situation cannot completely represent the prognosis of patients, which requires us to judge the disease of patients not only the initial state, but also the dynamic changes of the disease during treatment.

POD24 signifies poor prognosis across various diseases, including follicular lymphoma^5,10,11^. This concept has extended to other lymphomas with differing aggressiveness, indicating rapid progression and significantly reduced OS within 5 years^12–14^. While bortezomib-based treatment has significantly improved MM prognosis and extended PFS beyond 24 months, POD24 studies in MM remain scarce^15^.

This study demonstrates statistically significant differences in OS between patients with and without POD24, confirming its prognostic value in newly diagnosed MM patients. Furthermore, factor analysis identifies POD24 as an independent prognostic risk factor, introducing a novel parameter for MM prognosis. Unlike conventional baseline biological parameters, POD24 serves as an observation indicator at a fixed time point post-treatment initiation, allowing for past efficacy evaluation and early tailoring for future treatment strategies. Timely and accurate POD24 prediction and early identification of susceptible patients are crucial for improved MM prognosis. This study also identifies non-transplantation and chromosomal abnormalities as independent risk factors for POD24, providing valuable insights for distinguishing this subset of patients.

Current clinical MM prognostic models include ISS, R-ISS, and mSMART3.0. ISS primarily utilizes β2-MG and albumin for scoring, neglecting MM’s cytogenetic aberrations, a recognized prognostic factor. Consequently, its applicability in the new drug era is debated^16^. mSMART3.0 prioritizes stratified cytogenetic-based therapy, while R-ISS builds upon ISS by incorporating cytogenetics, resulting in greater applicability and advantages^17,18^. However, most patients fall under stage II, with heterogeneity in OS and PFS. R2-ISS and MASS staging address this by including 1q21 amplification and employing an integral scoring system, enabling finer classification of moderate-risk patients^2,3^.

Considering the clinical application of these models, this research investigated their efficacy in POD24 prediction. Our results indicate that mSMART3.0 displays higher sensitivity, while R-ISS has strengths in specificity and accuracy.Through the analysis of the ROC curve, it can be seen that the AUC value of each prognostic model is lower than 0.700, therefore, we believe that the existing MM prognostic models have poor predictive performance for POD24^21^. However, our results are only for reference and need to be verified with a larger sample size in the future.

In recent years, it has been suggested that 18 months after the start of treatment is an important time point for the evaluation of multiple myeloma^19^. Therefore, this study further divided the patients in the POD24 group into the POD18 group and compared to POD18-24. Survival analysis showed a significant difference in survival prognosis between the two groups, indicating that patients with disease progression or recurrence within 18 months had a worse prognosis. This is consistent with the recently proposed concept of functional high risk^20^.

Therefore, based on the above discussion, POD24 has practical prognostic value as an independent prognostic factor in MM, and seems to be relatively independent of existing prognostic models and can be used as a supplement to existing prognostic models. Patients with high-risk static parameters at onset can be considered to have a certain improvement in prognosis if there is no progression within 24 months after treatment, or patients defined as standard-risk group at onset have disease progression within 24 months, which can also be considered to have a poor prognosis. So, POD24 can be considered as a subsequent dynamic correction of static parameters at the beginning. In addition, treatment regimen is an important factor affecting prognosis. POD24 is a dynamic observation parameter during treatment, which is not limited by the initial treatment regimen and has certain advantages in prognosis judgment. In addition, the treatment plan can be adjusted in time according to POD24.

This study’s limitations include its retrospective nature with data from two centers. While the sample size of 473 is substantial, economic, and personal factors prevented some eligible patients from receiving bone marrow stem cell transplantation, leading to a limited transplant group and reduced generalizability. Additionally, the long follow-up period resulted in inconsistent treatment plans across patients. In the transplantation group, the treatment plan was relatively uniform, all patients were treated with PAD regimen for 4 courses of treatment followed by stem cell transplantation, and then treated with immunomodulator maintenance therapy. The treatment of non-transplant patients is relatively complicated due to various factors, but it is basically based on bortezomib. The inconsistency of treatment regimens leads to potential bias. Thirdly, due to the early follow-up time, the lack of popularity of FISH detection in the past, and the economic conditions of patients, most of the early patients were examined by traditional chromosome karyotype analysis. This may result in missing a subset of patients with chromosomal abnormalities. Finally, data on patients receiving newer drugs like CD38 monoclonal antibodies was absent, requiring further exploration.

## Conclusion

Based on the retrospective data mentioned above, it seemes that POD24 emerges as an independent poor prognostic factor for MM. Non-transplantation and chromosomal abnormalities further solidify its risk factors. According to our data, among the existing prognostic models, mSMART3.0 had the highest sensitivity in predicting POD24 and R-ISS had the highest specificity, but the AUC values of all models were less than 0.700, suggesting that there was no significant predictive efficacy. The need for a more accurate and timelier POD24 prognostic model remains unmet, particularly within the context of monoclonal antibody therapy not addressed in this bortezomib-based study. Future research should explore POD24 prognostic models and prediction in the era of new drug therapy to guide treatment adjustment and ultimately reduce POD24 occurrence and improve MM prognosis.

## Data Availability

The datasets generated during and/or analyzed during the current study are available from the corresponding author on reasonable request.
